# Ethnic differences in pregnancies complicated by gestational diabetes mellitus: a multiethnic cohort study

**DOI:** 10.1007/s12020-026-04624-5

**Published:** 2026-05-05

**Authors:** Kleoniki I. Athanasiadou, Georgios Markozannes, Fotini Kanouta, Marina Mitropoulou, Panagiotis Antsaklis, Kalliopi Pappa, Theodora Psaltopoulou, George Daskalakis, Dimitrios G. Goulis, Vasiliki Vasileiou, Stavroula A. Paschou

**Affiliations:** 1 Endocrine Unit and Diabetes Centre, Department of Clinical Therapeutics, Alexandra Hospital, School of Medicine, National and Kapodistrian University of Athens, 80 Vasilisis Sophias Avenue, Athens, 11528 Greece; 2 Department of Hygiene and Epidemiology, School of Medicine, University of Ioannina, University Campus, Ioannina, 45110 Greece; 3 Department of Endocrinology and Diabetes Centre, Alexandra Hospital, 80 Vasilisis Sophias Avenue, Athens, 11528 Greece; 4 1st Department of Obstetrics and Gynecology, Alexandra Hospital, National and Kapodistrian University of Athens, 80 Vasilisis Sophias Avenue, Athens, 11528 Greece; 5 Unit of Reproductive Endocrinology, 1st Department of Obstetrics and Gynaecology, Medical School, Aristotle University of Thessaloniki, Ring Road, Nea Efkarpia, 56403 Thessaloniki, Greece

**Keywords:** ethnicity, GDM, gestational diabetes mellitus, pregnancy

## Abstract

**Purpose:**

To identify ethnic differences among women with gestational diabetes mellitus (GDM) in a multiethnic cohort.

**Methods:**

This observational study included pregnant women with GDM (IADPSG criteria) attending a tertiary centre between January 2020-December 2023, classified according to their ethnic group as White, Black, Asian, or Other. Multivariable logistic and linear regression analyses were performed.

**Results:**

A total of 633 women (513 White, 64 Black, 32 Asian, and 24 Other/Middle Eastern) were considered eligible for inclusion. Asian women had higher HbA1c than White, Black, and Other (5.5% (37 mmol/mol) vs. 5.1% (32 mmol/mol), 5.2% (33 mmol/mol), and 5.1% (32 mmol/mol), respectively, *p* < 0.001). The mean birth weight was higher in Asian than White, Black, or Other groups (3429 g vs. 3102 g, 3279 g, and 3159 g, respectively, *p* = 0.05). Insulin therapy during pregnancy was more common in Asian and Other/Middle Eastern than White and Black women (71% and 79.2% vs. 54.5% and 52.4%, respectively, *p* = 0.03) and was independently associated with Middle Eastern ethnicity (aOR 6.73, 95% CI 1.31–34.46, *p* = 0.02).

**Conclusion:**

This study confirmed the presence of ethnic differences in pregnancies complicated by GDM and the higher metabolic burden in Asian and Middle Eastern populations. Ethnicity-specific strategies for GDM prevention and early screening should be implemented.

## Introduction

Gestational diabetes mellitus (GDM) is the most common metabolic complication of pregnancy and has several short- and long-term consequences that can adversely affect the mother and fetus. Women with a history of GDM have a 10-fold risk of developing type 2 diabetes compared to women with normoglycaemic pregnancies [1], while their offspring have an increased risk of developing obesity and metabolic syndrome in adolescence and adulthood [2, 3].

GDM affects approximately 14% of pregnant women, with the global prevalence ranging between 7% and 28% [4, 5]. The variation in its prevalence among different populations reflects ethnic origin as a potential risk factor for GDM development. Other GDM risk factors are obesity, advanced maternal age (> 35 years), history of GDM or macrosomia in a previous pregnancy, and family history of diabetes [6–8]. Early age at menarche and sleep disorders during pregnancy have been identified as potential risk factors [9, 10]. In some countries, screening for GDM is performed only in women with one of these risk factors, while other countries perform universal screening for GDM using an oral glucose tolerance test (OGTT). Among these risk factors, ethnicity presents the highest variability [11]. The risk of GDM is higher at a lower body mass index (BMI) threshold in some non-Caucasian populations, probably due to genetic predisposition, biological and socioeconomic factors [12]. The future risk of developing type 2 diabetes in women with GDM is also modified by ethnicity [13].

This study aimed to determine the phenotypical differences between women with GDM in a multiethnic cohort in a large tertiary centre, and to assess the variations in their pregnancy outcomes. To our knowledge, this is the first study evaluating the impact of ethnic differences on pregnancies complicated by GDM in the Greek population.

## Materials and methods

### Study characteristics

An observational, retrospective cohort study was performed including pregnant women with GDM and singleton pregnancies who attended the GDM outpatient clinic in a tertiary centre in Greece between January 2020 and December 2023. GDM was diagnosed with universal screening using the IADPSG criteria following a 2-hour 75-g OGTT performed approximately between the 24th and 28th gestational weeks. The glucose thresholds for GDM diagnosis were ≥ 92 mg/dl (5.1 mmol/l), ≥ 180 mg/dl (10 mmol/l), and ≥ 153 mg/dl (8.5 mmol/l) at 0, 60, and 120 min, respectively. At least one abnormal glucose value at any time point confirmed the diagnosis of GDM. During the study period, 646 women with GDM attended the GDM outpatient clinic of our tertiary centre. Ten women were excluded due to multiple gestation and three women due to non-declared ethnicity.

Women were classified into four categories according to their ethnic group as White, Black, Asian, or Other, using the 2021 Census of the United Kingdom for ethnicity classification [14]. White women were Greek, Greek-Cypriot, Albanian, Romanian, Bulgarian, or Georgian. Black women were of African or Caribbean descent. All Asian women originally came from South Asia and were Indian, Nepali, Pakistani, Bangladeshi, or Afghan. The group Other comprised women from Arab or Iranian heritage (Middle Eastern). Regarding their country of birth, all women in Black, Asian, and Other groups were reportedly foreign-born, whereas 411 (64.9%) of women in the White group were born in Greece. 

Prematurity was defined as delivery before the 37th gestational week, and macrosomia was defined as a birth weight of ≥ 4 kg. Large-for-gestational-age (LGA) was defined as birth weight ≥ 90th percentile for gestational age and sex and small-for-gestational-age (SGA) as a birth weight ≤ 10th percentile for gestational age and sex. All women received insulin as hypoglycaemic agent, as oral agents (metformin or glyburide) are not indicated for GDM management according to the Greek national clinical practice guidelines, which are followed in our tertiary centre [15]. The criteria for insulin treatment initiation included: (a) persistently (several times weekly) elevated fasting glucose concentrations [above 95 mg/dl (5.3 mmol/L)] or postprandial glucose concentrations [(above 140 mg/dl (7.8 mmol/L) at 1 h postprandially)] despite intensive medical nutrition therapy and regular exercise (as long as there was no obstetric contraindication), (b) asymmetric fetal macrosomia (defined as fetal abdominal circumference > 75th percentile), and (c) polyhydramnios (defined as the vertical measurement of the deepest pocket of amniotic fluid free of fetal parts ≥ 8 cm or the amniotic fluid index (AFI) ≥ 24 cm).

### Statistical analysis

Data were expressed as mean ± standard deviation (SD) for continuous variables and as absolute numbers and percentages (%) for categorical variables. Multivariable linear and logistic regression analyses were performed to determine the association between ethnicity and the following outcomes: insulin therapy during pregnancy, polyhydramnios, labour induction, caesarean section, perinatal injury, Apgar score at 5 min, prematurity, neonatal birth weight, gestational age at delivery, LGA, SGA, and neonatal macrosomia. The results were expressed as odds ratios (OR) with a 95% confidence interval (95% CI), with a significance level of 5%. Formal analysis was performed using Stata V16.1. Maternal age, BMI at OGTT, HbA1c at OGTT, history of GDM in previous pregnancy, history of macrosomia in previous pregnancy, family history of diabetes, polycystic ovary syndrome (PCOS), smoking during or before pregnancy, thyroid disorders (pre-existing hypothyroidism, gestational hypothyroidism, and hyperthyroidism), and conception via in vitro fertilization/assisted reproductive technology (IVF/ART) were the potential confounders for which the models were adjusted for.

## Results

A total of 633 women, including 513 (81%) White, 64 (10.1%) Black, 32 (5.1%) Asian, and 24 (3.8%) Other, were included in the study. Of them, 271 delivered in the obstetric unit of the same tertiary centre and their pregnancy outcomes were assessed, while the rest were either lost to follow-up, opted to deliver in a private obstetric service, and one miscarried. All pregnancies were singleton and led to live births. The distribution of live births per ethnic group was: 227 (83.8%) in White, 24 (8.9%) in Black, 12 (4.4%) in Asian, and 8 (3%) in Other.

**Fig. 1:**
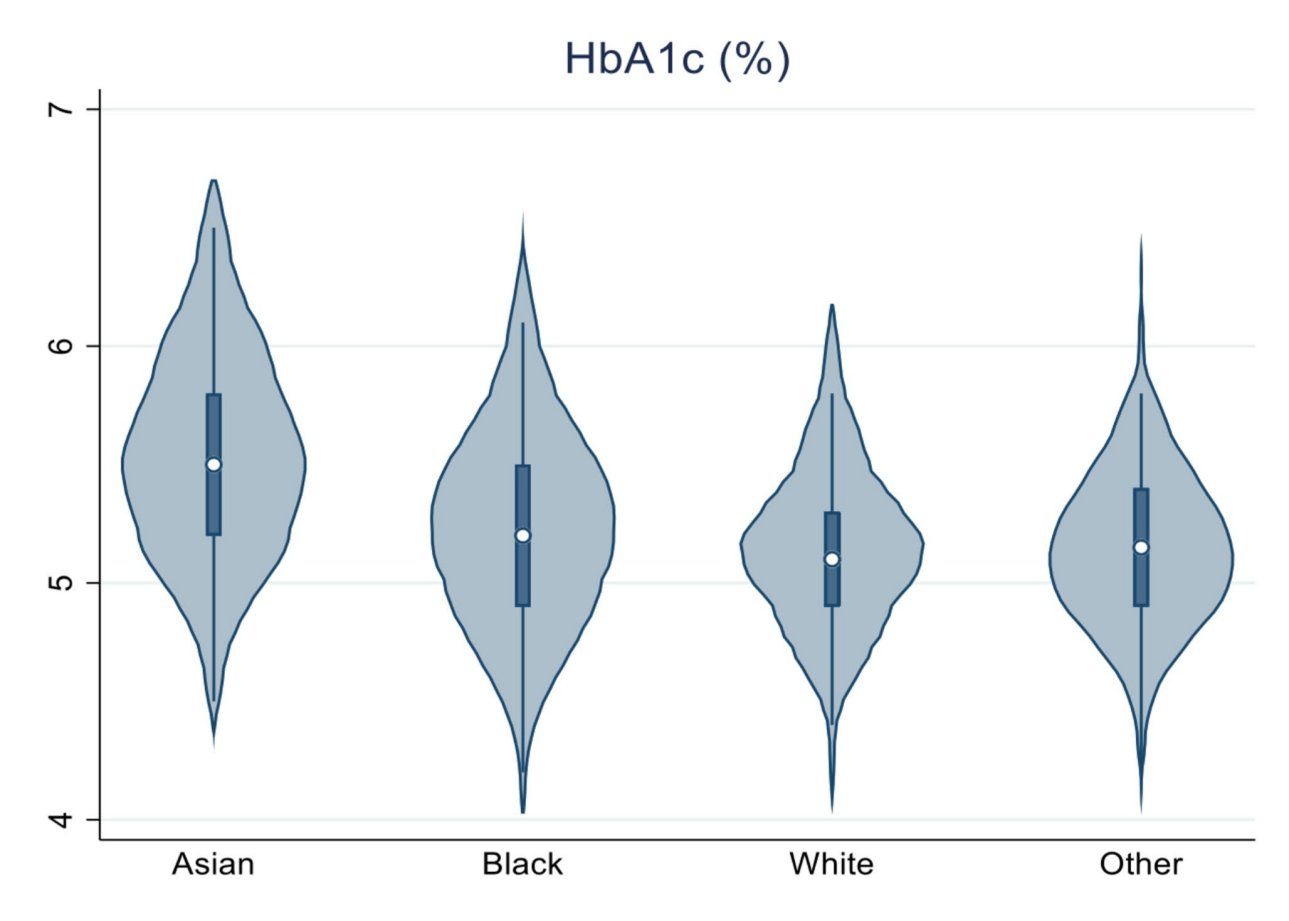
Violin plot illustrating differences in the HbA1c concentrations among ethnic groups at the time of the OGTT

Notably, while no statistically significant difference was observed, Asian women had the lowest BMI compared to White, Black, and Other groups both pre-pregnancy (25.7 ± 3.6 kg/m^2^ vs. 27.1 ± 6.7 kg/m^2^, 28 ± 5.9 kg/m^2^, and 28.1 ± 5.6 kg/m^2^, respectively, *p* = 0.574) and at OGTT (28.3 ± 4.2 kg/m^2^ vs. 30.2 ± 6.3 kg/m^2^, 30.6 ± 5 kg/m^2^, and 30.4 ± 4.9 kg/m^2^, respectively, *p* = 0.525). Despite that they had statistically significant higher HbA1c compared to the other groups (5.5% (37 mmol/mol) vs. 5.1% (32 mmol/mol), 5.2% (33 mmol/mol), and 5.1% (32 mmol/mol), respectively, *p* < 0.001, Fig. 1). Family history of diabetes was numerically (but not statistically) higher in White and Asian groups compared to Black and Other groups (30.7% and 29% vs. 25% and 16.7%, respectively, *p* = 0.413). Maternal age, gestational weight gain, and age at menarche were similar across the groups. PCOS was more prevalent in White women than in Black, Asian, and Other groups (6.1% vs. 3.2%, 3.2%, and 0%, respectively, *p* = 0.435). Congenital malformations (*n* = 3) were observed only in the White group.

**Fig. 2:**
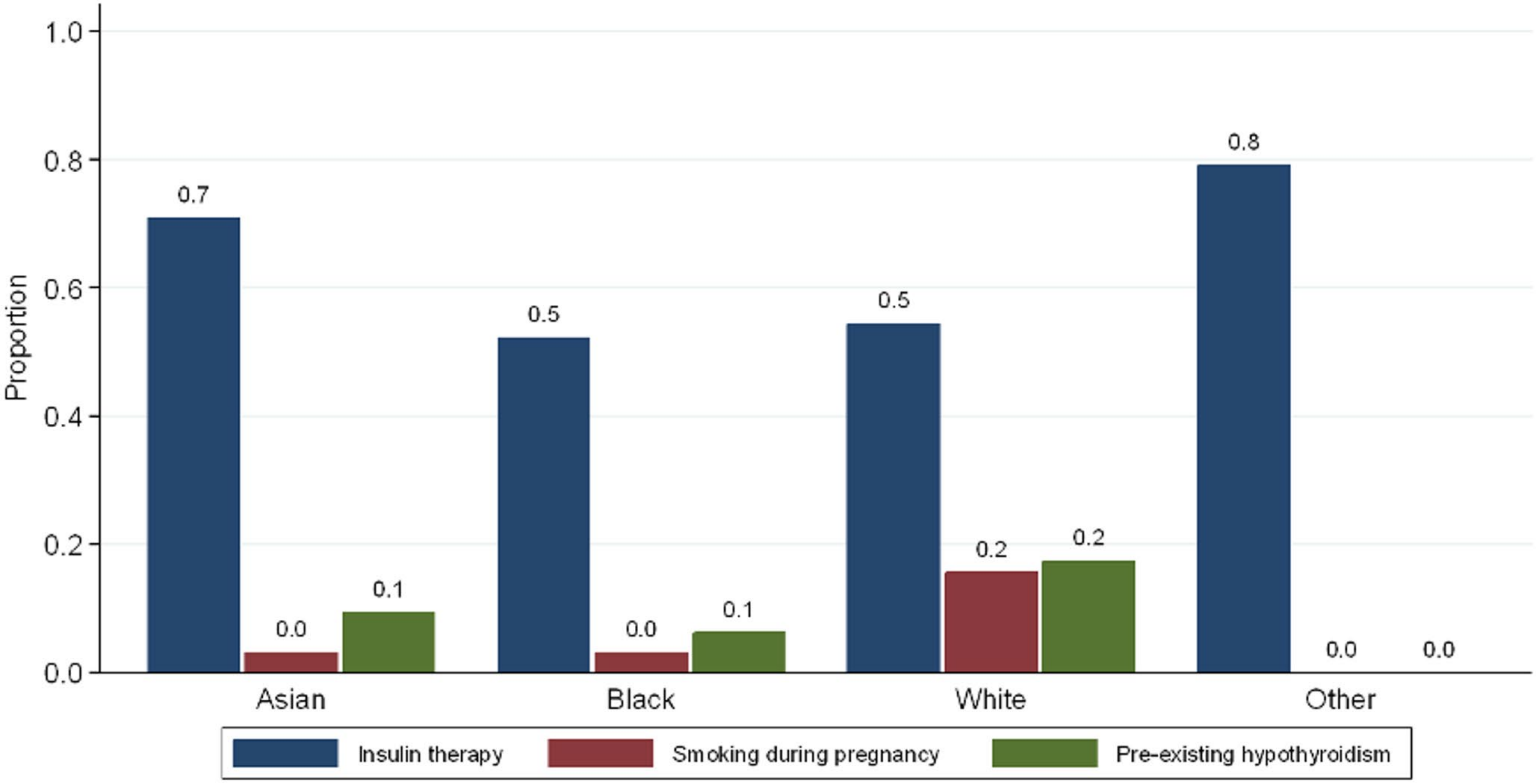
Baseline differences in categorical variables among ethnic groups

**Fig. 3:**
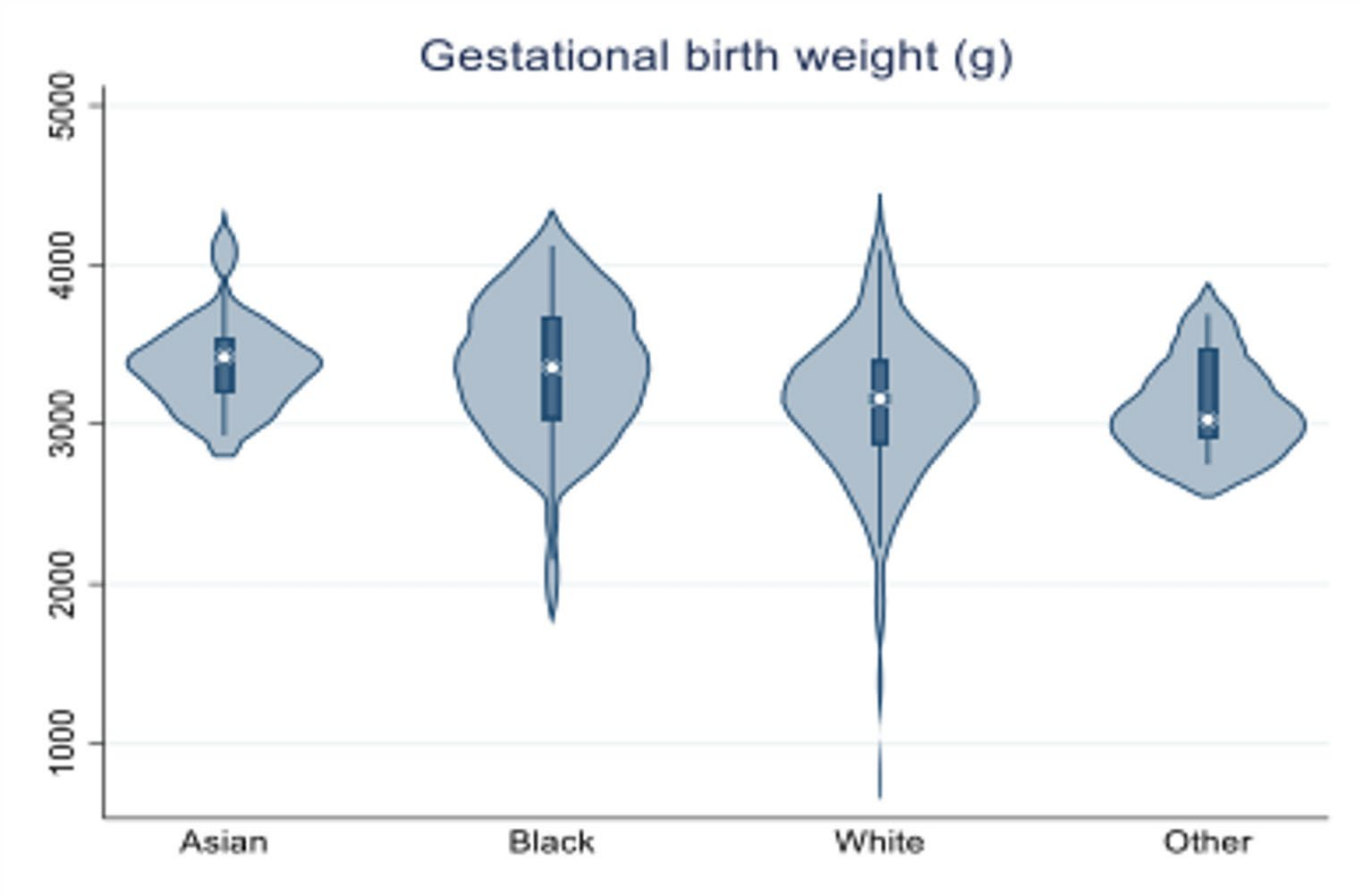
Violin plot illustrating differences in the neonatal birth weight among ethnic groups

In addition, insulin requirements during pregnancy were significantly higher in Asian and Other groups compared to White and Black (71% and 79.2% vs. 54.5% and 52.4%, respectively, *p* = 0.03, Fig. 2). Pre-existing hypothyroidism was more prevalent in White women compared to Black, Asian, and Other groups (17.4% vs. 6.3%, 9.4%, and 0%, respectively, *p* = 0.01, Fig. 2). However, gestational/pregnancy-induced hypothyroidism was more prevalent in Black women compared to White, Asian, and Other groups (15.6% vs. 9.2%, 9.4%, and 12.5%, respectively, *p* = 0.424) but without statistically significant difference. Interestingly, the percentage of women who were ex-smokers was significantly higher in White compared to the Asian, Black, and Other groups (9.4% vs. 3.1%, 1.6%, and 0%, respectively *p* = 0.04). The difference was even higher for White women who were actively smoking during pregnancy (15.7% vs. 3.1%, 3.1%, and 0%, respectively, *p* = 0.002, Fig. 2). The baseline characteristics of all ethnic groups are summarized in Table 1. The pregnancy outcomes of the different ethnic groups are summarized in Table 2. Regarding pregnancy outcomes, neonatal birth weight was higher in the Asian group compared to White, Black, and Other [(3429 ± 368 g) vs. (3102 ± 488 g), (3279 ± 526 g), and (3159 ± 352 g), respectively, *p* = 0.05, Fig. 3].

**Table 1:** Baseline characteristics between ethnic groups

	White*n* = 513 (81.0%)	Black*n* = 64 (10.1%)	Asian*n* = 32 (5.1%)	Other*n* = 24 (3.8%)	Total*n* = 633 (100.0%)	*p*-value
Maternal age (years)	33.1 (6.8)	33.3 (6.3)	33 (5.7)	31.9 (6.3)	33.1 (6.7)	0.835
<18y 18-34y >35y	9 (1.8) 256 (49.9) 248 (48.3)	1 (1.6) 32 (50.0) 31 (48.4)	0 (0.0) 19 (59.4) 13 (40.6)	0 (0.0) 16 (66.7) 8 (33.3)	10 (1.6) 323 (51.0) 300 (47.4)	0.658
**Age at menarche (years)**	12.8 (1.6)	13.4 (1.5)	12.5 (1.3)	12.8 (1.9)	12.8 (1.6)	0.623
**Pre-pregnancy BMI (kg/m** ^**2**^ **)**	27.1 (6.7)	28.0 (5.9)	25.7 (3.6)	28.1 (5.6)	27.2 (6.5)	0.574
**BMI at OGTT (kg/m** ^**2**^ **)**	30.2 (6.3)	30.6 (5.0)	28.3 (4.2)	30.4 (4.9)	30.2 (6.1)	0.525
**Weight gain (kg)**	7.9 (6.9)	7.1 (8.5)	6.5 (4.7)	8 (8.4)	7.8 (7.0)	0.708
**Gestational week at OGTT**	26.3 (4.6)	27.5 (3.0)	25.3 (5.0)	25.1 (5.3)	26.3 (4.5)	0.422
**HbA1c** % mmol/mol	5.1 (0.4) 32 (5)	5.2 (0.4) 33 (5)	5.5 (0.5) 37 (5.5)	5.1 (0.4) 32 (5)	5.1 (0.4) 32 (5)	< 0.001
**Conception via IVF/ART**						
No	500 (97.7)	64 (100.0)	30 (93.8)	24 (100.0)	618 (97.8)	
Yes	12 (2.3)	0 (0.0)	2 (6.3)	0 (0.0)	14 (2.2)	0.218
**PCOS**						
No	478 (93.9)	61 (96.8)	30 (96.8)	24 (100.0)	593 (94.6)	
Yes	31 (6.1)	2 (3.2)	1 (3.2)	0 (0.0)	34 (5.4)	0.435
**Family history of diabetes**						
No	355 (69.3)	48 (75.0)	22 (71.0)	20 (83.3)	445 (70.5)	
Yes	157 (30.7)	16 (25.0)	9 (29.0)	4 (16.7)	186 (29.5)	0.413
**History of GDM**						
No	405 (79.4)	46 (71.9)	23 (74.2)	19 (82.6)	493 (78.5)	
Yes	105 (20.6)	18 (28.1)	8 (25.8)	4 (17.4)	135 (21.5)	0.478
**History of macrosomia**						
No	483 (94.5)	55 (85.9)	31 (96.9)	22 (91.7)	591 (93.7)	
Yes	28 (5.5)	9 (14.1)	1 (3.1)	2 (8.3)	40 (6.3)	0.051
**History of preeclampsia**						
No	489 (96.1)	63 (98.4)	31 (96.9)	24 (100.0)	607 (96.5)	
Yes	20 (3.9)	1 (1.6)	1 (3.1)	0 (0.0)	22 (3.5)	0.599
**Smoking during pregnancy**						
No	431 (84.3)	62 (96.9)	31 (96.9)	24 (100.0)	548 (86.8)	
Yes	80 (15.7)	2 (3.1)	1 (3.1)	0 (0.0)	83 (13.2)	0.002
**Ex-smoker before pregnancy**						
No	463 (90.6)	63 (98.4)	31 (96.9)	24 (100.0)	581 (92.1)	
Yes	48 (9.4)	1 (1.6)	1 (3.1)	0 (0.0)	50 (7.9)	0.043
**Gestational hypothyroidism**						
No	464 (90.8)	54 (84.4)	29 (90.6)	21 (87.5)	568 (90.0)	
Yes	47 (9.2)	10 (15.6)	3 (9.4)	3 (12.5)	63 (10.0)	0.424
**Pre-existing hypothyroidism**						
No	422 (82.6)	60 (93.8)	29 (90.6)	24 (100.0)	535 (84.8)	
Yes	89 (17.4)	4 (6.3)	3 (9.4)	0 (0.0)	96 (15.2)	0.011
**Pre-existing hyperthyroidism**						
No	508 (99.4)	63 (98.4)	32 (100.0)	24 (100.0)	627 (99.4)	
Yes	3 (0.6)	1 (1.6)	0 (0.0)	0 (0.0)	4 (0.6)	0.741

Data are expressed as mean (standard deviation, SD) for continuous variables and as n (%) for categorical variables

**Abbreviations**: BMI: body mass index; GDM: gestational diabetes mellitus; HbA_1c_: glycated haemoglobin; IVF/ART: in vitro fertilization/assisted reproductive technology; OGTT: oral glucose tolerance test; PCOS: polycystic ovary syndrome; y: years

**Table 2:** Pregnancy outcomes between ethnic groups

	White*n* = 227 (83.8%)	Black*n* = 24 (8.9%)	Asian*n* = 12 (4.4%)	Other*n* = 8 (3.0%)	Total*n* = 271 (100.0%)	*p*-value
**Insulin therapy during pregnancy**						
No	230 (45.5)	30 (47.6)	9 (29.0)	5 (20.8)	274 (43.9)	
Yes	276 (54.5)	33 (52.4)	22 (71.0)	19 (79.2)	350 (56.1)	0.032
**Vaginal delivery**						
No	165 (72.7)	18 (75.0)	10 (83.3)	4 (50.0)	197 (72.7)	
Yes	62 (27.3)	6 (25.0)	2 (16.7)	4 (50.0)	74 (27.3)	0.420
**Induction of labour**						
No	202 (89.0)	20 (83.3)	11 (91.7)	7 (87.5)	240 (88.6)	
Yes	25 (11.0)	4 (16.7)	1 (8.3)	1 (12.5)	31 (11.4)	0.847
**Caesarean section**						
No	62 (27.3)	6 (25.0)	2 (16.7)	4 (50.0)	74 (27.3)	
Yes	165 (72.7)	18 (75.0)	10 (83.3)	4 (50.0)	197 (72.7)	0.420
**Pregnancy-induced hypertension**						
No	212 (93.4)	22 (91.7)	12 (100.0)	8 (100.0)	254 (93.7)	
Yes	15 (6.6)	2 (8.3)	0 (0.0)	0 (0.0)	17 (6.3)	0.670
**Preeclampsia**						
No	220 (99.1)	24 (100.0)	12 (100.0)	8 (100.0)	264 (99.2)	
Yes	2 (0.9)	0 (0.0)	0 (0.0)	0 (0.0)	2 (0.8)	0.940
**Polyhydramnios**						
No	198 (89.2)	21 (87.5)	9 (75.0)	7 (87.5)	235 (88.3)	
Yes	24 (10.8)	3 (12.5)	3 (25.0)	1 (12.5)	31 (11.7)	0.522
**Prematurity**						
No	187 (82.7)	21 (87.5)	11 (91.7)	6 (75.0)	225 (83.3)	
Yes	39 (17.3)	3 (12.5)	1 (8.3)	2 (25.0)	45 (16.7)	0.716
**Gestational age at delivery (weeks)**	37.7 (1.8)	38.2 (2.0)	38.0 (0.9)	38.1 (1.3)	37.8 (1.8)	0.613
**Neonatal birth weight (g)**	3102 (488)	3279 (526)	3429 (368)	3159 (352)	3134 (488)	0.057
**LGA**						
No	215 (95.1)	23 (95.8)	10 (83.3)	8 (100.0)	256 (94.8)	
Yes	11 (4.9)	1 (4.2)	2 (16.7)	0 (0.0)	14 (5.2)	0.289
**SGA**						
No	210 (92.9)	22 (95.7)	12 (100.0)	8 (100.0)	252 (93.7)	
Yes	16 (7.1)	1 (4.3)	0 (0.0)	0 (0.0)	17 (6.3)	0.632
**Neonatal macrosomia**						
No	220 (97.3)	23 (95.8)	11 (91.7)	8 (100.0)	262 (97.0)	
Yes	6 (2.7)	1 (4.2)	1 (8.3)	0 (0.0)	8 (3.0)	0.650
**NICU admission**						
No	209 (92.9)	22 (91.7)	12 (100.0)	8 (100.0)	251 (93.3)	
Yes	16 (7.1)	2 (8.3)	0 (0.0)	0 (0.0)	18 (6.7)	0.659
**Congenital malformations**						
No	208 (98.6)	23 (100.0)	12 (100.0)	8 (100.0)	251 (98.8)	
Yes	3 (1.4)	0 (0.0)	0 (0.0)	0 (0.0)	3 (1.2)	0.892
**Perinatal injury**						
No	188 (83.6)	21 (87.5)	11 (91.7)	6 (75.0)	226 (84.0)	
Yes	37 (16.4)	3 (12.5)	1 (8.3)	2 (25.0)	43 (16.0)	0.739
**Apgar score (5 min)**	8.5 (0.7)	8.2 (0.7)	8.3 (0.9)	8.3 (0.7)	8.4 (0.7)	0.229

Data are expressed as mean (standard deviation, SD) for continuous variables and as n (%) for categorical variables

**Abbreviations**: LGA: Large for gestational age; NICU: neonatal intensive care unit; SGA: Small for gestational age

In the multivariable analyses, two different models were run, with (*n* = 441) and without (*n* = 633) adjustment for BMI and HbA1c due to missing values. Middle Eastern ethnicity (group: Other) was independently associated with an increased risk of insulin therapy during pregnancy (aOR 3.18, 95% CI 1.13–8.97, *p* = 0.03). When the model was adjusted for BMI and HbA1c the risk was even higher (aOR 6.73, 95% CI 1.31–34.46, *p* = 0.02). Asian ethnicity was also associated with an increased risk of insulin therapy requirement; however, the result was not significant (aOR 2.03, 95% CI 0.88–4.66, *p* = 0.10) and was attenuated after adjustment for BMI and HbA1c (aOR 0.95, 95% CI 0.31–2.85, *p* = 0.92). Additionally, a history of GDM or macrosomia in a previous pregnancy was independently associated with insulin therapy during pregnancy [(aOR 2.09, 95% CI 1.35–3.24, *p* < 0.01) and (aOR 2.53, 95% CI 1.11–5.75, *p* = 0.03), respectively] across all ethnicities. After adjusting for BMI and HbA1c the association remained significant for the history of GDM (aOR 2.05, 95% CI 1.17–3.58, *p* = 0.01) but not for the history of macrosomia (aOR 1.31, 95% CI 0.49–3.54, *p* = 0.59). There were no significant differences in the other outcomes between groups in the two models, even after combining LGA, SGA, and macrosomia in one composite outcome (fetal growth abnormalities). The multivariable analysis results can be found in the supplementary material.

## Discussion

The present study aimed to determine the phenotypic differences between women with GDM from a multiethnic cohort and evaluate the effect of ethnicity on their pregnancy outcomes. An important finding was the discrepancy between the lower BMI but higher HbA1c, neonatal birth weight, and insulin requirement during pregnancy observed in Asian women compared to the other ethnic groups. This finding highlights the increased metabolic risk in people of Asian heritage which has been described in the literature and is generally attributed to biological/genetic and socioeconomic factors [16, 17]. In light of that, the World Health Organization (WHO) has proposed lower thresholds for overweight and obesity in Asian populations at 23 kg/m^2^ and 27.5 kg/m^2^, respectively [18].

All Asian women in our cohort came from South Asia, which has been described as one of the ethnic regions with the highest GDM prevalence, presenting at lower BMI levels compared to the general population [19]. South Asian ethnicity has also been associated with a high early GDM (eGDM) risk (presenting before the 20th gestational week) in the large clinical trial TOBOGM (Treatment of Booking Gestational Diabetes Mellitus) [20]. Importantly, the study showed that the risk for eGDM remained elevated even after adjustment for socioeconomic factors (aOR 1.29, 95% CI 1.04–1.59, *p* < 0.001). Another interesting association has been found between ethnicity, socioeconomic status, and pregnancy outcomes in women with GDM. South Asian ethnicity has been associated with an increased future risk of developing type 2 diabetes but a lower risk of developing depression compared to White individuals [21].

Our study results are in line with previous findings of similar original studies showing a higher risk of GDM and higher insulin resistance in women of Middle Eastern origin compared to women of Caucasian origin [22]. The significant degree of heterogeneity characterizing GDM derives from ethnic differences and the unique metabolic fingerprint of various ethnic groups [23–25]. In a previous study evaluating the different GDM subtypes according to the 2-hour 75-g OGTT results, it was shown that Asian ethnicity was more common in the groups of isolated fasting and combined hyperglycaemia which were the two groups related to higher insulin resistance and unfavourable pregnancy outcomes compared to the group of isolated postprandial hyperglycaemia [26].

In addition, several studies have shown the advantages of implementing ethnicity-specific BMI thresholds in clinical practice, providing earlier intervention in ethnic minorities, who present higher metabolic and pregnancy complications at lower BMI values [27–29]. In our study, the mean BMI across ethnic groups were > 25 kg/m^2^ indicating that most women in the cohort were overweight or obese, highlighting maternal weight as a major modifiable GDM risk factor [30]. The establishment of structured weight management services in preconception care could enhance women’s efforts to achieve a healthy pre-pregnancy weight using ethnicity-specific BMI thresholds, targeting to GDM prevention or lowering the risk of GDM development/recurrence [31]. Additionally, the observed differences in smoking habits with a predominance of White ethnicity in both ex-smokers and active smokers during pregnancy seem to reflect the cultural variations between ethnic groups. Notably, in the Middle Eastern group (Other), the percentage of smoking before or during pregnancy was 0%. In a recent systematic review and meta-analysis of thirty-five studies, smoking during pregnancy was not associated with an increased risk of GDM [32]. However, considering the high risks of placental dysfunction, preeclampsia, stillbirth, preterm delivery, and congenital anomalies, prompt referral to smoking cessation services must become a prenatal priority for women of reproductive age [33].

In other studies, the predominance of non-White ethnicity (Middle Eastern and Asian) on the outcome of increased insulin requirement during pregnancy is explained using the theory of the “nutrition transition” from women’s traditional diet to a more calorie-dense Western diet [34]. However, this epigenetic explanation is not fully applicable to our study population, as the Mediterranean diet, which is the main established diet followed in Greece, is mostly plant-based and one of the healthiest worldwide [35]. Of course, we acknowledge that dietary patterns and habits can be directly impacted by ethnicity, and people may prefer to maintain their traditional diet/cuisine. In such cases, the applicability of “nutrition transition” theory is limited. Considering the absence of official assessment tools to evaluate the maternal diet during pregnancy, we would suggest the close monitoring of maternal diet in GDM pregnancies by providing individualized dietary plans [36]. It is common practice in our service to discuss and advise on maternal diet at every clinic appointment to improve the quality of their meals, focusing on a low glycaemic index, and provide tailored advice aiming to improve their glycaemic control and avoid insulin treatment, if possible.

Our study presents both strengths and limitations that should be acknowledged. From the strengths perspective, the sample size of our study was large and ethnically diverse. Considering the universal screening for GDM in all pregnant women in Greece and our centre being one of the leading referral centres for diabetes in pregnancy in the country, we support that the sample reflects the GDM population in a national level; therefore, elimination of selection bias has been achieved. In Greece, more than 90% of the population is White, therefore women in non-White groups constitute ethnic minorities. Interestingly, all women in our cohort had a mean time of OGTT within the 24th -28th gestational weeks’ timeframe and were offered regular appointments in clinic to closely monitor their glycaemic and blood pressure control. Interpreter services were also available in clinic to mitigate language barriers. The single-centre and retrospective format, the small size of the Black, Asian, and Other groups compared to White individuals, and the lack of a comparator group with normoglycaemic women are the study’s limitations. The high percentage of women who were followed up in the GDM clinic but did not deliver in our hospital’s obstetric unit and the smaller size of non-White groups compared to White group may have limited the power to detect associations in the multivariable analysis of pregnancy outcomes.

The study has important research and clinical implications. Women from the Asian and Middle Eastern ethnic groups presented the highest metabolic risk compared to women from the White and Black groups. Considering that ethnicity is not a modifiable risk factor, we would suggest prioritizing earlier GDM screening before the 24th gestational week in these women aiming for early GDM diagnosis and prompt lifestyle and medical interventions. Earlier GDM screening with an OGTT could be considered in women of high-risk ethnicities in countries with multi-ethnicity composition where universal GDM screening has not been established. The differences among ethnic groups indicate that women belonging to ethnic minorities must be under close antenatal follow-up. The application of advanced telemedicine systems and continuous glucose monitoring (CGM) can reduce the number of face-to-face appointments, intensify diabetes care, and increase the effectiveness of GDM management [37, 38].

In conclusion, this study highlights the ethnic differences among women with GDM in a diverse, multiethnic cohort in Greece. The differences in baseline characteristics and pregnancy outcomes across ethnic groups indicate the need to implement tailored strategies focusing on GDM prevention and early GDM screening to mitigate antenatal and postnatal risks.

## Data Availability

The data that support the findings of this study are not openly available due to reasons of sensitivity and are available from the corresponding author upon reasonable request.
